# Synthesizing Molecular and Immune Characteristics to Move Beyond WHO Grade in Meningiomas: A Focused Review

**DOI:** 10.3389/fonc.2022.892004

**Published:** 2022-05-31

**Authors:** Nivedha V. Kannapadi, Pavan P. Shah, Dimitrios Mathios, Christopher M. Jackson

**Affiliations:** Department of Neurosurgery, Johns Hopkins University School of Medicine, Baltimore, MD, United States

**Keywords:** meningioma, immunotherapy, immune microenvironment, prognostic model, risk score, meningioma WHO grade I

## Abstract

No portion of this manuscript has previously been presented. Meningiomas, the most common primary intracranial tumors, are histologically categorized by the World Health Organization (WHO) grading system. While higher WHO grade is generally associated with poor clinical outcomes, a significant subset of grade I tumors recur or progress, indicating a need for more reliable models of meningioma behavior. Several groups have developed risk scores based on molecular or immunologic characteristics. These classification schemes show promise, with several models preliminarily demonstrating similar or superior accuracy to WHO grading. Improved understanding of immune system recognition and targeting of meningioma subtypes is necessary to advance the predictive power, as well as develop new therapies. Here, we characterize meningioma molecular drivers, predictive of recurrence and progression, and describe specific aspects of the immune response to meningiomas while highlighting critical questions and ongoing research. Relevant manuscripts of interest were identified using a systematic approach and synthesized into this focused review. Finally, we summarize the ongoing and completed clinical trials for immunotherapy in meningiomas and offer perspective on future directions.

## Introduction

Meningiomas are the most common primary intracranial neoplasm, comprising approximately 40% of all primary central nervous system tumors (1). Treatment for symptomatic or enlarging tumors consists of maximum safe surgical resection with radiation therapy applied to residual, recurrent, or high-risk lesions (2). There are no effective chemotherapy regimens for meningiomas and clinical trials for targeted therapies are ongoing (3). Meningiomas are classified according to World Health Organization (WHO) grade, with the majority (69-78%) classified as grade I, followed by grades II and III (20.4-30% and 1-1.6%, respectively) (4, 5). Grades II and III tumors recur more frequently, with rates varying between 28-52% and 40-84%, respectively (6–9). The degree of surgical resection correlates with the risk of tumor recurrence or progression and is classified by the Simpson grading scale (10). However, many tumors recur despite aggressive resection. Simpson grade 1 involves gross total resection of the tumor with removal of affected dura and bone and has a 5-year recurrence rate ranging from 0 to 21% (11). Grade 2, which involves gross total resection with cautery of dural margin, and grade 3, gross total resection, are associated with 5-year recurrence rates of 5-33% and 0-40%, respectively (11). Subtotal resection, classified as grade 4, is associated with a 5- and 10-year recurrence rate of 49-52% and 83%, respectively (12, 13). While higher WHO grades are associated with worse clinical outcomes, a grade I designation does not reliably predict tumor behavior, as 10-47% of grade I meningiomas recur or progress (14, 15).

WHO grade-matched meningiomas exhibit considerable heterogeneity, particularly in genetic alterations and immune cell infiltration. This diversity implies that a molecular and inflammatory classification framework might ultimately prove superior to the WHO grading system for predicting meningioma behavior. Predictive risk scores based on these factors have shown promise in identifying both high-risk grade I tumors and low-risk grade III tumors. One such molecular classification system defined four unique consensus molecular phenotypes and found that an “immunogenic” phenotype was associated with improved clinical outcomes (16). These findings indicate that a detailed understanding of how the immune system recognizes and attacks specific meningioma subtypes could more reliably predict progression.

In this focused review, we consider meningioma tumor characteristics, such as mutations, chromosomal aberrations, and hypermethylation, that may carry prognostic value independent of WHO grade. We also discuss specific aspects of the immune response to meningiomas, including tumor-intrinsic factors such as mutational burden as well as the mechanics of immune recognition and tumor cell elimination. Finally, we synthesize the available data and highlight some of the necessary steps to develop a molecular and immunologic classification scheme for meningiomas.

## Methods

We systematically reviewed the relevant literature on molecular and immune environments of meningiomas, published on Pubmed from database inception to April 2022. We identified 750 studies, using the search string “(Meningioma AND immunology) OR (Meningioma AND chemokine) OR (Meningioma AND molecular AND classification)”. Included studies must (1) describe meningiomas and (2) include characterization of immunologic or molecular disease features. Exclusion criteria include (1) non-English primary language (2) abstract only with no full-text manuscript (3) studies of other brain tumors with no data on meningiomas and (4) no discussion of clinical implications of molecular/immune markers. 66 manuscripts were included after abstract screening, and 40 manuscripts were included after full text review. Remaining studies of interest were accessed based on the reference lists of these manuscripts.

## Molecular Drivers of Meningioma Recurrence and Progression

Meningiomas have several key molecular alterations that have been extensively described (17, 18). The 2021 WHO classification criteria incorporates 10 genes that are frequently altered in meningiomas, including *NF2, AKT1, TRAF7, SMO, PIK3CA* (19). Additionally, *TERT* promoter mutations have been significantly associated with progressive recurrence in grade I meningiomas (20). Since 40-60% of sporadic meningiomas have a loss of *NF2* expression, meningiomas can be molecularly categorized into *NF2* mutants and non-*NF2* mutants, with the latter predominantly comprised of *TRAF7*, *KLF4*, *AKT1*, *SMO*, and *P13K* mutations (17, 18). Some of these molecular biomarkers have been independently associated with meningioma progression and recurrence (21, 22). In low grade meningiomas, Youngblood et al. established a link between mutations in the *HH* and *TRAF7* genes and increased recurrence rates (23). Recently, a number of studies have demonstrated the prognostic implications of PI3K/AKT/mTOR pathway mutations (24–26). One study of over 80 samples found that an activating *AKT1* mutation, upstream of mTOR, was associated with recurrence and present in 32% of grade I tumors (27). Mutations in the *SMO* gene have also been associated with poor prognosis in grade I meningiomas (28). In a study of over 500 patients, Sievers et al. found that meningiomas with *CDKN2A/B* homozygous deletions had a significantly shorter time to progression or recurrence (29)

Several studies have also revealed that chromosomal aberrations, such as 1p/14q codeletion, correlate with recurrence (30–33). In one study of mostly grade I orbital meningiomas, progressive tumors were more likely to have loss of chromosome 1p and 6q (34). Specific metabolite concentrations within tumor tissue have also been associated with clinical outcome. Pfisterer et al. used magnetic resonance spectroscopy to demonstrate that higher glycine to glutamine/glutamate ratio, glutamine to glutamate ratio, and creatine concentration is associated with rapidly recurring meningiomas (35). Another study characterized a highly metabolically active subgroup of benign meningiomas, linked to mutations in genes regulating transcription and metabolism, and found this subgroup to be associated with increased recurrence rates (36).

Radiation also has an important effect on the molecular background and immune environment. Agnihotri et al. compared the mutational and methylation profiles of radiation-induced meningiomas, as a result of childhood radiotherapy to the brain, with those of sporadic meningiomas. Radiation-induced meningiomas were associated with structural rearrangements of the *NF2* gene, loss of chromosomes 1p and 22q, and decreased focal gene mutations that are characteristic of non-*NF2* tumors (37). Using whole genome sequencing, RNA sequencing, and ChIP sequencing for histone H3K27ac, Paramasivam et al. identified important differences between sporadic meningiomas and those arising after radiation (38). This study showed that although both sporadic and radiation-induced meningiomas have features of homologous recombination repair (HRR) failure, the underlying cause varies, with the former exhibiting greater genomic instability resulting in deficiency of HRR genes and the later having exhausted HRR at sites of radiation-induced DNA damage.

Characterizing the embryological origins of the meninges has elucidated location-specific meningioma biomarkers that show promise as prognostic indicators (39). Kalamarides et al. characterize prostaglandin D synthase-positive meningioma precursor cells and the embryonic window when mutations in these cells cause meningioma development with distinct genetic signatures (40). Using over 250 meningioma cases, Okano et al. found that tumors originating from the paraxial mesoderm were associated with mutations in *AKT*, *KLF4*, *SMO*, and *POLR2A*, whereas neural crest-derived meningiomas were associated with *NF2* mutations (41). Additionally, this group identified *POLR2A* mutations as a risk factor for recurrence.

The field of radiomics, which typically uses machine learning to correlate quantitative imaging characteristics with tumor features, has emerged as a promising, noninvasive method of studying the molecular characteristics of tumors. While several studies use imaging to accurately predict clinical outcome in meningiomas (42, 43), researchers have only recently examined the associations between imaging and the molecular landscape of meningiomas. One study of 314 meningioma samples identified specific radiologic features that predicted recurrence and overall survival and found that tumors with certain features were more likely to have higher somatic mutational burden, DNA methylation, and expression of the pro-mitotic transcription factor *FOXM1* (44). In a prediction model using over 60 grade II meningiomas, Shin et al. found that lower apparent diffusion coefficient 10^th^ percentile was an independent predictor of *TERT* promoter mutation (45). Although the current understanding of meningioma radiomics is in its nascency, the field shows great promise for diagnostic and therapeutic application.

Recent efforts have made strides towards synthesizing molecular risk factors into predictive models. Schmidt et al. conducted a transcriptomic analysis that identified 8 differentially expressed genes, including *PTTG1* and *LEPR* which were associated with poor prognosis (46). Another group used microarray transcriptomics to cluster meningiomas into three prognostic groups, showing that *CKS2*, *UBE2C*, and *TFPI2* were associated with recurrence (47). Patel et al. generated a transcriptomics-based grading system which was superior WHO grade in predicting recurrence. This study found that the group associated with the highest recurrence rate typically showed loss of the DREAM complex, which regulates the cell cycle (48). Chen et al. identified a 36-gene signature characteristic of clinically aggressive meningiomas and developed a risk score that reliably predicted recurrence and survival (49). This risk score was significantly associated with overall survival, whereas WHO grade was not, suggesting increased prediction accuracy. Dai et al. identified over 1600 differentially expressed genes, enriched in PI3K-Akt signaling pathways, extracellular matrix organization, and cytokine-chemokine receptor interactions, among others (50). While this group did not develop a prediction model for recurrence based on these enriched pathways, further characterization of these genes in aggressive meningiomas shows promise for improved classification schemes.

Epigenetic factors correlate with meningioma recurrence and progression. Loss of trimethylated histone H3K27 has been associated with recurrence of grades I and II tumors (51) (52) (53). One study described a 49-gene expression signature associated with high-risk tumors and found that hypermethylation of genes associated with cell-cycle regulation and the WNT signaling pathway, such as UCHL1 and SFRP1, predicted aggressive behavior (54). Using 140 meningioma samples, Olar et al. established a 64-CpG loci methylation predictor to categorize tumors into two prognostic groups, which were independently associated with recurrence (55). Both Sahm et al.’s classification into 6 prognostic groups based on methylation of 40 genes and Nassiri et al.’s methylome-based model more accurately predict 5-year recurrence-free survival than clinical factors and WHO grade (56, 57). Recently, Maas et al. developed a molecular-morphologic score, based on histology, copy-number variation, and methylation class, that significantly outperformed WHO grading in predicting recurrence (58). Berghoff et al. defined three prognostic methylation clusters, based on 126 meningioma samples, that are significantly better at predicting PFS than WHO grade is (22). Synthesizing DNA methylation, RNA sequencing, and cytogenetic data, Baylev et al. classified meningiomas into three biological groups with unique signatures and distinct prognoses (59). These efforts demonstrate the potential superiority of molecular classification schemes over histologic classification in predicting meningioma outcomes.

## Immune Signatures of Meningiomas

**Figure 1:**
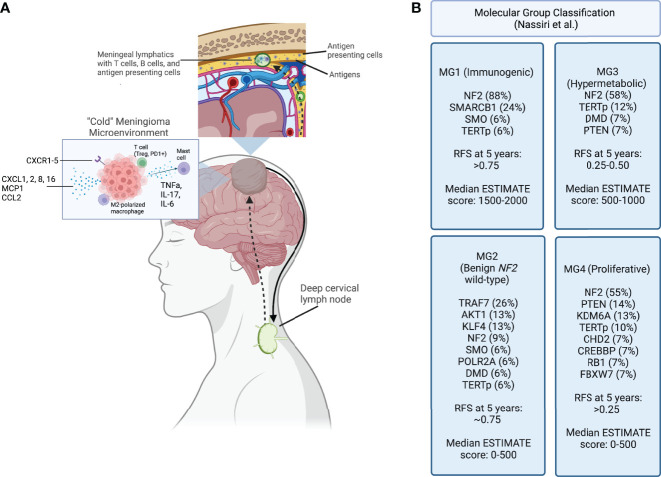
**(A)** Meningioma tumor antigens are taken up by antigen presenting cells and carried to the meningeal lymphatic system, which drain to the deep cervical lymph node. After antigen presentation and immune activation occurs in the lymph node, immune cells are trafficked to the tumor site. A “cold” meningioma microenvironment consists of immunoregulatory cytokines, and immunosuppressive cell populations such as M2 polarized macrophages and regulatory T cells. **(B)** Molecular classification schema developed using a multimodal characterization approach by Nassiri et al., including the most frequently point-mutated genes within each category. Recurrence-free survival (RFS) at 5 years and the ESTIMATE score, quantifying immune infiltration based on expression of genes, are reported for each group. Created with BioRender.com.

Alongside advances in molecular characterization, there has been recent progress in understanding the immune composition of meningiomas (**Figure 1**). The meningioma immune microenvironment is mostly comprised of macrophages, T cells, and mast cells. Macrophages are the predominant immune cell in meningiomas (60–62). Proctor et al. found that tumor-associated macrophages account for almost 20% of all cells in meningioma tissues, and that M2 polarization is associated with recurrence (63, 64). An increase in peripheral myeloid-derived suppressor cells (MDSCs) and programmed death ligand 1 (PD-L1)+ monocytes is seen in patients with high-grade meningiomas (65). Interestingly, a study of patients with grade I and II meningiomas described expansion of peripheral “MDSC-like” monocytes, with MDSC markers but no T cell suppressive activity, and increased functional MDSCs in tumor samples, suggesting the importance of MDSC induction at the tumor site (66). A study of 40 brain-invasive meningiomas found that 25% had macrophages or microglia at the tumor-brain interface (67). These findings suggest a central role for macrophages in driving meningioma-immune cell interactions.

Cytotoxicity of tumor-infiltrating lymphocytes is essential to immune responses against tumor cells. Regulatory T cells, programmed cell death protein 1 (PD-1)+ CD8+ cells, and PD-1+ CD4+ cells are associated with high-grade and aggressive tumors (65, 68, 69). High density of regulatory T cells are also a prognostic marker for recurrence (68, 70). In a study of over 200 high-grade meningiomas, Rapp et al. report that increased cytotoxic tumor-infiltrating lymphocytes are associated with prolonged PFS (71). Tumor location may further play a role in T cell infiltration. Comparing skull base to convexity meningiomas, Zador et al. found increased activity of oncolytic gamma-delta T cells in grade I skull base tumors (72). On the other hand, cavernous sinus meningiomas exhibit decreased immune cell infiltration, including cytotoxic lymphocytes, regulatory T cells, and tumor-associated macrophages (TAMs), as compared to convexity meningiomas (73). While this study does not independently correlate these immune findings to outcomes, further characterization of site-specific immune microenvironments may better predict the role for immunotherapy based on location and elucidate fundamental mechanisms of immune cell egress into specific intracranial compartments.

In a systemic review of studies evaluating the meningioma immune environment, mast cells were found to be present in up to 90% of meningiomas classified as grades 2 and 3 and were primarily located in perivascular areas (74). Peritumoral edema was also correlated with mast cell infiltration. Another study of secretory meningiomas, comprising 1.1% of meningiomas at this institution, showed increased mast cell infiltration and edema when compared to non-secretory tumors (75). Although this subtype is rare and it is not clear whether mast cell presence is causative, the presence of mast cells may be leveraged as a prognostic tool. In a study of grade I meningiomas, convexity tumors were more associated with the presence of activated mast cells compared with skull base tumors, and cytokine-cell networks showed mast cells were most strongly correlated with IL-6 (72).

Other important cells present in the tumor microenvironment include dendritic cells. Interestingly, when Chen et al. used CIBERSORT technology in 68 meningioma samples to characterize the immune environment, increased dendritic cells were found to be significantly associated with poor prognosis. Samples were categorized as “high” or “low” dendritic cell count based on the median content of cells, and differentially expressed genes were identified in each group. The B cell receptor signaling pathway was found to be activated in the “low” group, indicating increased presence of B cells in these groups. The authors hypothesized that increased dendritic cells may directly or indirectly reduce B cell infiltration in meningiomas, leading to worse outcome (76).

Alterations in antigen presentation appear to play an important role in meningioma biology. Tumor mutations lead to generation of neoantigens that can be processed by antigen presenting cells (APCs) and loaded onto major histocompatibility complex (MHC) molecules. Both soluble neo-antigens as well as APCs can travel *via* the meningeal lymph vessels to the deep cervical lymph nodes (77). Because meningiomas are extra-axial and have varying degrees of brain invasion, the relative contributions of brain-specific vs head and neck lymphatics in driving antigen availability is unclear. Particularly, it is important to determine the contribution of the glymphatic system, which is characterized by the exchange of cerebrospinal fluid and parenchymal interstitial fluid with eventual drainage into the deep cervical lymph nodes *via* narrow periarterial channels (78). Of potential importance, lymphatic vessels located in the meninges are much wider than these periarterial channels. Accordingly, the APCs presenting antigens could more readily drain through the meninges vs. periarterial channels, which may be limited to soluble antigens (79). Additionally, T cells, B cells, and dendritic cells are present in meningeal lymphatics and participate in immune surveillance (80). Lastly, studies describing the effects of meningiomas on the cerebral vasculature have shown increased permeability to proteins (81). As such, it is possible that meningiomas are not protected by the full complement of immunoregulatory mechanisms in the CNS and, therefore, may be more susceptible to immune clearance compared with other brain tumors once an adequate immune response has been initiated.

The link between the glymphatic and meningeal lymphatic systems is poorly understood. To-date there are no studies examining the implications of these systems on antigen presentation in meningiomas. As data emerge on this topic, it may be of particular interest to characterize how proximity of the tumor to lymphatic vessels, degree of immune surveillance, and vascular permeability affect prognosis. Although T cell migration from peripheral lymph nodes to meningioma tissue has yet to be explored directly, extrapolating data from other brain tumors and CNS inflammatory conditions it is likely that antigen-educated lymphocytes travel *via* the afferent meningeal lymphatic vessels. Immune cells then home to brain tumors by chemokine stimulation (82). It remains to be shown which pathways mediate meningioma antigen-specific responses. However, meningiomas have been found to express several cytokines and chemokines, indicating that they are immunologically active (83, 84). Barbieri et al. found that at least one of the CXC receptors 1-5 was constitutively expressed in over 75% of meningiomas (85). Expression of CX3CL1, a chemokine that mediates migration of T cells, dendritic cells, and natural killer (NK) cells, positively correlates with tumor grade, whereas CXCL16, a T cell and monocyte chemoattractant, has increased expression in grade I samples (86). In a study characterizing differentially expressed genes between meninges and atypical meningiomas, Cao et al. found that CXCL2 and CXCL8 levels were not only upregulated in tumor tissue, but also independently associated with overall survival and recurrence (87). Another group investigated the role of monocyte chemoattractant protein-1 (MCP-1) in meningiomas and found high expression and a positive correlation between MCP-1 expression and macrophage infiltration (88). Similarly, CCL2, a monocyte chemoattractant, was found to be highly expressed in meningioma tissue (89). Cytokine-cytokine receptor interaction networks have already been developed to inform prognostic scoring of meningioma samples (72, 90). A consistent finding of these studies is that the cytokine milieu is extraordinarily heterogenous between tumors, and some have posited that ligand/receptor imbalance may impact tumor progression (86). Taken together, these data suggest that a predictive model accounting for the interactions between immune cells and meningiomas could have a prognostic potential.

## Interactions Between Molecular Patterns and Immune Signatures

Tumor mutational patterns influence the composition of the immune microenvironment. Broadly, increased mutational burden has been reported in progressive and high-grade meningiomas and may correlate with inflammation (91). Gill et al. described more peritumoral edema in patients with increased single nucleotide variants (92) and higher tumor mutational burden is associated with increased immune cell infiltration (33). Rutland et al. evaluated 145 meningioma samples and found that a scattered distribution of lymphocytes was associated with increased point mutations (93). In a phase II clinical trial of 25 patients receiving nivolumab for grade II/III meningiomas, patients with higher mutational burden were more likely to respond to immunotherapy (94). The patient with the longest recurrence-free survival was also deficient in *MSH2*, a DNA mismatch repair gene (95). These studies provide evidence that, as in many other neoplasms, availability of high-quality antigens is a driver of immune responses against meningiomas.

While overall mutational burden may influence immune infiltration, several specific mutational patterns have related to unique immune signatures. Williams et al. described three molecular patterns of high-grade and progressive grade I meningiomas, and the majority of these 850 aggressive tumors were classified as NF2-mutant (96). NF2-mutated grade I tumors have a higher density of M2 macrophage infiltration than that of tumors with AKT1 activating mutations, suggesting that this genetic subset of grade I meningiomas may drive immunosuppression. Alternatively, AKT1 activation may also cause M1 polarization (97). M1 macrophages, along with NK cells and recently activated lymphocytes, are also enriched in tumors with chromosome 22 monosomy, indicating that genes on chromosome 22 may be closely linked to immunosuppression (98). Genetic alterations in meningiomas have been linked to activity of immune checkpoint pathways. Among non-NF2-mutated tumors, TRAF7 and AKT1 mutations are associated with expression of PD-L1, IDO, and TDO2 (99). Tumors with SMO and PIK3CA mutations have been linked to cytotoxic T-lymphocyte-associated protein 4 (CTLA-4)+ lymphocyte infiltration (100). One study found a significant association between DNA polymerase epsilon mutations and CD8+ infiltration as well as improved PFS (101). Mast cell infiltration has also been correlated with specific molecular drivers: Xie et al. developed a risk score by analyzing differentially expressed genes related to resting mast cells, immune cell abundance, miRNA-mRNA co-expression network, and drug-gene interaction prediction. Importantly, the 9 key genes identified were important in the signaling of TNF-alpha, IL-17, and other cytokines, supporting the importance of further elucidating the interplay between molecular and immunologic signatures (90).

Several groups have used data processing tools to further characterize how the molecular tumor signature impacts the immune environment. Nassiri et al. recently performed a molecular analysis of 124 meningiomas of various locations and histological subtypes that included DNA sequencing, DNA methylation, RNA expression and single cell RNA sequencing. They reported 4 distinct molecular subgroups based on an integrative analysis of multi-platform genomic and epigenomic data. One of the subgroups (MG1) was characterized by expression of several immune pathways. This group of patients had recurrent NF2 mutations and loss of chromosomal arm 22q that resulted in biallelic inactivation of the *NF2* gene. Interestingly, this group was significantly enriched in T cell and macrophage genes and demonstrated the highest levels of cytokine and immune checkpoint molecule expression among the subgroups. This group of patients also exhibited the longest recurrence free survival. In contrast, subgroup MG4 was characterized by expression programs that allow for higher proliferation of tumor cells and contained the fewest macrophage-associated genes. This group had the highest recurrence rate compared to the other groups as well as the highest tumor mutational burden. While the latter finding contrasts with some previous reports, this data indicates that proliferation of tumor cells may outpace immunologic clearance. If this is the case, this subgroup may have a higher response rate to immune checkpoint inhibitors (16). Chen et al. created two clusters based on high versus low expression of RNA methylation regulators (m^6^A regulators) and correlated the groups to immune infiltration. There were significant differences between infiltration of plasma B cells, resting mast cells, and neutrophils, as well as expression of IL-15 and IL-18 (102). These findings suggest that incorporating the presence of specific mutations into a predictive model may have value and guide implementation of immunotherapy.

PD-L1 is one of the most frequently studied checkpoint molecules in meningioma. Several studies have shown that PD-L1 expression is independently correlated with worse clinical outcome (103, 104). Additionally, Karimi et al. found that co-expression of PD-L1 with hypoxia-induced genes, such as NFKB2 and CA9, correlates with tumor progression (105). While these studies have established an association between PD-L1 expression and tumor progression, the use of PD-L1 alone as a biomarker may be limited by generally infrequent expression. Johnson et al. reported PD-L1 positivity values of 3%, 6%, and 18% for grades I, II, and III, respectively (106). The Tumor Immunity in the MicroEnvironment (TIME) scale, which categorizes tumors according to PD-L1 and tumor-infiltrating lymphocyte (TIL) positivity, has been implemented to predict responses to immunotherapy in several types of tumors (107). Yeung et al. used multiplex quantitative immunofluorescence to classify 73 meningiomas according to the TIME scale and found that most fell into the poor responder groups of PD-L1^low^TIL^low^ and PD-L1^low^TIL^high^. Notably, PD-L1 was more highly expressed on CD68+ macrophages than tumor cells, and PD-L2 was more strongly associated with T cell proliferation and cytotoxicity than PD-L1 (108). Both PD-L2 and B7-H3 expression have been associated with mutations of the mTOR pathway, including PI3K, AKT1, and mTOR (100). Interestingly, this study found PD-L2 to be most enriched in grade I meningiomas. Taken together, these findings indicate that PD-L2 and B7-H3 may play a more central role in meningiomas and, accordingly, PD-1 blockade may have activity in PD-L1 negative tumors. Based on the emerging relationship between tumor genomics and the immune microenvironment, further exploration of genomic alterations and immune-based risk predictors is warranted.

## Clinical Studies

Clinical implementation of immunotherapy for meningiomas is still in its nascency. Early studies investigating the use of interferon alpha for recurrent meningiomas produced negative results (109). One retrospective case series of patients with recurrent grade II or III meningiomas treated with interferon alpha noted no radiographic responses at first evaluation and progression free survival was 17% at 6 months (110). A phase 2 study investigating interferon alpha for recurrent grade I intracranial meningiomas also reported no neuroradiographic responses, and PFS at 6 months was 54% (111).

Immune checkpoint inhibition has shown anecdotal promise and is currently being evaluated in clinical trials. Two case reports have been published describing the use of nivolumab for recurrent meningiomas. A case report of a patient on nivolumab for advanced lung cancer who also had recurrent right sphenoid wing meningioma reported significant reduction in both tumor size and brain edema following initiation of therapy (112). Another report described a response to checkpoint blockade in a MSH2-deficient tumor. After therapy the patient had a marked increase in CD8+ T cell infiltration of the tumor. The patient continued to receive nivolumab bi-weekly for over 2 years and experienced a marked response (95).

Based on these early reports, there are now several ongoing clinical trials aimed at evaluating checkpoint blockade for meningiomas (**Table 1**). While three of the trials are recruiting patients for anti-PD1 monotherapy, most studies include at least one treatment arm investigating combination therapy regimens. Given Han et al.’s findings that patients who received radiation have higher expression of PD-L1, four trials are investigating the synergy between immunotherapies targeting the PD-1/PD-L1 axis and various types of radiation therapy (103). In a phase II study of the anti-PD1 agent pembrolizumab, patients have improved PFS rates at 6 months, compared to historical controls, and a non-significant association between increased PD-L1 expression and reduced tumor growth (113). Additionally, two trials include treatments targeting the CD28-CTLA-4 pathway. While nivolumab with or without ipilimumab therapy in recurrent atypical meningiomas does not show improvement in 6 month PFS, a subset of tumors with increased mutational burden may have higher response rates (94, 95). Successful completion of these trials will provide valuable insights into the clinical utility of immune checkpoint blockade for meningiomas.

**Table 1:** Active clinical trials assessing immunotherapy for meningiomas registered on ClinicalTrials.gov.

Identifier	Status	Intervention	Study design	Estimated Enrollment	Patient cohort	Summary of published data
**NCT03173950**	Recruiting	Nivolumab	Phase 2; Non-Randomized, Parallel Assignment	180	Grade II or III; recurrent	Not applicable
**NCT02648997**	Recruiting	Nivolumab ± ipilimumab and external beam radiation therapy	Phase 2; Non-Randomized, Sequential Assignment	50	Grade II or III; recurrent	Well tolerated but no improvement in progression-free survival (94) Responder to therapy shows high tumor mutational burden (95)
**NCT03604978**	Recruiting	Nivolumab and radiosurgery ± ipilimumab	Phase 1/2; Randomized, Parallel Assignment	15	Grade II or III; recurrent	Not applicable
**NCT03016091**	Recruiting	Pembrolizumab	Phase 2; Single Group Assignment	25	Grade II or III; recurrent	Not applicable
**NCT04659811**	Recruiting	Pembrolizumab and radiosurgery	Phase 2; Non-Randomized, Parallel Assignment	90	Grade I, II, or III; recurrent	Not applicable
**NCT03267836**	Recruiting	Avelumab and proton beam radiotherapy	Phase 1; Single Group Assignment	12	Grade I, II, or III; recurrent	Not applicable
**NCT03279692**	Active, not recruiting	Pembrolizumab	Phase 2; Single Group Assignment	26	Grade II or III; recurrent or residual	Improved progression-free survival at 6 monthsTrend between increased PD-L1 expression and decreased tumor growth (113)

## Conclusion

Ongoing work focused on tumor-immune interactions has the potential to afford valuable insights into the drivers of meningioma behavior. The link between tumor cell alterations and the immune landscape is particularly intriguing intersection. Although immune-based characterization of meningiomas has only recently garnered interest, ongoing efforts in this area will drive more robust prediction models and new therapeutic strategies for patients with recurrent and progressive tumors.

## Author Contributions

NK, PS, DM, and CJ contributed to conception and design of the review. NK and PS wrote the first draft of the manuscript. DM and CJ wrote sections of the manuscript. All authors contributed to manuscript revision, read, and approved the submitted version.

## Funding

Meningioma research in CJ’s laboratory is supported by the Goldhirsh-Yellin Foundation, Grant number (138405).

## Conflict of Interest

CJ is the co-founder and owns equity interest in Egret Therapeutics. This arrangement has been reviewed and approved by the Johns Hopkins University in accordance with its conflict of interest policies.

The remaining authors declare that the research was conducted in the absence of any commercial or financial relationships that could be construed as a potential conflict of interest.

## Publisher’s Note

All claims expressed in this article are solely those of the authors and do not necessarily represent those of their affiliated organizations, or those of the publisher, the editors and the reviewers. Any product that may be evaluated in this article, or claim that may be made by its manufacturer, is not guaranteed or endorsed by the publisher.
